# Evaluating and Visualizing the Contribution of ECG Characteristic Waveforms for PPG-Based Blood Pressure Estimation

**DOI:** 10.3390/mi13091438

**Published:** 2022-08-31

**Authors:** Gang Ma, Yuhang Chen, Wenliang Zhu, Lesong Zheng, Hui Tang, Yong Yu, Lirong Wang

**Affiliations:** 1 School of Biomedical Engineering (Suzhou), Division of Life Sciences and Medicine, University of Science and Technology of China, Hefei 230026, China; 2 Suzhou Institute of Biomedical Engineering and Technology, China Academy of Sciences, Suzhou 215163, China; 3 School of Electronics and Information Technology, Soochow University, Suzhou 215006, China

**Keywords:** ECG characteristic waveforms, Grad-CAM, PPG, CNN, blood pressure

## Abstract

Non-invasive continuous blood pressure monitoring is of great significance for the preventing, diagnosing, and treating of cardiovascular diseases (CVDs). Studies have demonstrated that photoplethysmogram (PPG) and electrocardiogram (ECG) signals can effectively and continuously predict blood pressure (BP). However, most of the BP estimation models focus on the waveform features of the PPG signal, while the peak value of R-wave in ECG is only used as a time reference, and few references investigated the ECG waveforms. This paper aims to evaluate the influence of three characteristic waveforms in ECG on the improvement of BP estimation. PPG is the primary signal, and five input combinations are formed by adding ECG, P wave, QRS complex, T wave, and none. We employ five common convolutional neural networks (CNN) to validate the consistency of the contribution. Meanwhile, with the visualization of Gradient-weighted class activation mapping (Grad-CAM), we generate the heat maps and further visualize the distribution of CNN’s attention to each waveform of PPG and ECG. The heat maps show that networks pay more attention to the QRS complex and T wave. In the comparison results, the QRS complex and T wave have more contribution to minimizing errors than P wave. By separately adding P wave, QRS complex, and T wave, the average MAE of these networks reaches 7.87 mmHg, 6.57 mmHg, and 6.21 mmHg for systolic blood pressure (SBP), and 4.27 mmHg, 3.65 mmHg, and 3.73 mmHg, respectively, for diastolic blood pressure (DBP). The results of the experiment show that QRS complex and T wave deserves more attention and feature extraction like PPG waveform features in the continuous BP estimation.

## 1. Introduction

Hypertension is one of the most serious but potential public health problems in the world, which will threaten the patients’ life and can even result in many diseases such as heart failure, stroke, coronary heart disease, etc. [1]. Modern medical evidence shows that early detection and strict control of hypertension could slow its further development and reduce the risk of disease [2]. Nevertheless, as it is often referred to as a “silent killer”, most people with pre-hypertension have no significant symptoms and always ignore it. *The Lancet* reports that the number of people with hypertension between the ages of 30 and 79 doubled from 1990 to 2019, and nearly 580 million people are unaware of it [3]. Therefore, continuous BP monitoring is an effective and necessary solution for the early detection and control of these diseases [4].

In general, there are two basic kinds of BP measurement methods: direct and indirect. Direct measurement, as the gold standard, is carried out by the insertion of a catheter, but is often restricted in critical situations such as intensive care units and clinical surgeries; its result is the most accurate and continuous in nature [5]. As this method is invasive and carries multiple risks, clinically people tend to use indirect methods. Traditionally they use an inflatable cuff to compress the blood vessel and observe changes in pulse wave signal during this process. However, the measurement mechanism of inflation and deflation is cumbersome and not suitable for long-term observation [6]. What is more, due to the pressure on blood vessels, the time interval between two monitoring readings needs to be greater than 2 min. In the past decade, with the emergence of wearable devices that are convenient for physiological signal collection [7], continuous blood pressure monitoring has gradually shown a lot of promise. Based on PPG and ECG, the researchers summarized three basic physiological indicators highly related to BP: pulse arrival time (PAT), pulse transit time (PTT) [8,9], and pulse wave velocity (PWV) [10]. Many studies have proved the effectiveness of these three indicators [11]. Once the PTT, PAT, or PWV are determined, BP can be calculated from a mathematical model.

The above three indicators are essentially based on the blood flow velocity in the blood vessel, and another research trend is the shape of the waveform. The propagation of pulse waves along the artery is affected by heart, vascular consistency, vascular stiffness, vascular resistance [12], etc. Changes in these factors will also be reflected in the waveform shape. Therefore, researchers focus on morphology and are keen to extract characteristic features from these signals [13,14]. Kruylyak [15] extracted 21 physiological indices from PPG features and used an artificial neural network (ANN) model to verify the improvement in accuracy. Liu et al. [16] extracted 14 feature parameters from the second derivative of the PPG(SDPPG) and combined 21 regular time-domain PPG features to establish a Support Vector Regression (SVM). In addition to using PPG and ECG signal features, Yin [17] also added personal information, such as age, weight, and gender from 186 volunteers. From PPG and its derivatives, Lin et al. [18] proposed 19 new physiological parameters and the predicted BP value achieved a decrease when tested on 22 subjects. In the literature [19], the author combined up to 107 features from each PPG signal, including 75 time-domain, 16 frequency-domain and 10 statistical features, as well as 6 demographic data. Then, the feature selection technique was used to reduce the amount of computation, and the Gaussian Process Regression (GPR) method is used. The estimation performance of the ReliefF feature selection algorithm outperformed other algorithms in estimating. The authors above have made full use of PPG waveform characteristics, while only a few articles discussed ECG features. To the best of our knowledge, Jamal [20] firstly explored the importance of the ECG waveform in a BP model. They applied the CNNs to extract 56 features from PPG and ECG segments. By adding and removing ECG features, they proved that ECG waveforms contained important information which is helpful to improve accuracy. Then Geerthy [21] compared the model trained with PPG features fused with 18 ECG features and PPG features alone. They investigated the influence of these features and used random forest-based genetic algorithm (GA) and confirmed that significant features like QRS complex, QT interval, SDI, and heart rate could improve the results.

Compared with classical machine learning models, neural network models have gradually been applied to the field of continuous BP monitoring based on physiological signals and have become a new trend. This method eliminates the step of manual feature extraction and takes advantage of its powerful data mining and feature extraction capabilities [22]. In [23], a four-layer long short-term memory (LSTM) network was employed to estimate systolic blood pressure (SBP) and diastolic blood pressure (DBP). The model contains a bidirectional structure for accessing larger-scale context information of input sequence and residual connections to allow efficient gradient in the LSTM network to propagate. Tanveer et al. [24] combined the preprocessed PPG and ECG waveforms into the hierarchical ANN-LSTM to obtain the value of SBP and DBP. Yu et al. [25] improved U-Net with an attention-based residual and added derivatives of the PPG signal as additional inputs, aiming to improve the effectiveness of information mining. Rong [26] proposed a multi-type features fusion (MTFF) neural network model. This model contains two basic blocks: one is CNN block, which was used to learn the morphological and frequency spectrum features, and the other is Bi-directional LSTM (BLSTM) block, which was used to focus on temporal features.

To sum up, it can be found that BP prediction models based on physiological signals are the mainstream trend of continuous BP monitoring. On the one hand, researchers focus on the extraction of new effective artificial features. However, most of the literature pays too much attention to the characteristics of PPG signal, with the R-peak of the ECG signal used as the time reference point, while few articles use the characteristics of ECG waveform. On the other hand, the CNN models have been continuously improved to extract features, and ECG and PPG signals are used as the original signal input. However, due to the uninterpretable nature of neural networks, it is difficult for people to know which feature of the signal the networks have learned.

In order to better understand and explain CNNs, a visualization technique known as Grad-CAM [27] was developed for visual interpretation. It utilizes gradient information flowing into the last convolutional layer of the CNNs to assign importance values to each neuron for a specific attention decision. Grad-CAM is an improvement of CAM [28] that overcomes the problem inherent in CAM. The obvious shortcoming of CAM is that the network structure needs to be modified, which means that the data need to be retrained. Grad-CAM solved this problem and is suitable for any CNN-based network without modifying the structure. Li et al. [29] applied Grad-CAM to the channel selection of Electroencephalography (EEG) signal and achieved great accuracy. Kin et al. [30] used Grad-CAM for the interpretation of the basis of the judgment of the ECG classification model. They proposed a visual-DenseNet for ECG model using Grad-CAM. These experiments show that Grad-CAM is also suitable for 1-dimensional signals.

Finally, the main contributions of this study are listed as follows:

(1) This paper specifically compares the contribution of the three characteristic waveforms of ECG to the BP prediction model. Each ECG signal is masked as separate P, QRS, and T waves. Then these signals are put into different networks combined with PPG signal for training, rather than directly adding or deleting whole ECG signals [20].
(2) This paper introduces Grad-CAM into a BP regression model so that we can more clearly understand which features of physiological signals will receive more attention or be ignored in the model, so as to provide a reference for artificial feature extraction or modification of the network model. Meanwhile, the visualization results further corroborate with (1), which strongly illustrate the different characteristics that ECG signals and PPG signals provide in the model.

## 2. Materials and Methods

### 2.1. Dataset

In this study, original PPG, II-lead ECG, and continuous arterial blood pressure (ABP) signals are from the MIMIC-III database [31]. MIMIC-III is an extensive, freely available database comprising health-related data from more than 40,000 patients who stayed in critical care units of the Beth Israel Deaconess Medical Center between 2001 and 2012 [32]. It contains thousands of distinct physiological signal records and vital sign time series collected from bedside patient monitors in the adult and neonatal intensive care unit, and the sampling rate is 125 Hz. Due to human operation factors, these records can last from a few seconds (usually abnormal) to several hours. For details of the MIMIC-III database, see https://mimic.physionet.org/ accessed on 13 January 2021. PPG and ECG signals are inputs, and the ABP signals are used for calculating the SBP and DBP values as the reference values in this experiment.

### 2.2. Preprocessing

#### 2.2.1. Signal Selection and Filtering

Since the whole database is very large and the signal quality is even, we conduct a detailed cleaning procedure by the following criteria: First, we used raw PPG and ECG waveforms as network input, and ABP was used to make labels. Some records may lack one of these signals. Second, each waveform needs to be long enough to contain some changes in SBP and DBP. The minimal required record length was set to 1 min. Third, due to occasional human activity that led to sensor separation, a flat line appeared for a period of time in some records, which caused the loss of valid data [33]. Fourth, some records may contain abnormal ABP values (e.g., SBP ≥ 180 or DBP ≤ 60) [34]. Once a record has any of the above conditions, the corresponding record will be removed. The final database retained 938 subject records.

In order to remove the impacts of noise and artifacts during the collection process, some filters were further implemented to denoise the signals. PPG is susceptible to baseline drift, motion artifacts, and high-frequency noise, so a 4th order Butterworth bandpass filter with cutoff frequencies of 0.05 and 10 Hz was used to remove interfering signals [35]. For ECG, we used a 3rd order Butterworth bandpass filter with cutoff frequencies of 0.05 and 35 Hz [21]. Then each filtered signal was cut into 8 s fragments. In order to make blood pressure labels, we conducted peak detection on ABP signals, taking the mean peak value of each fragment as SBP and the valley value as DBP. The final dataset consists of 9280 fragments and is split into the training set, validating set, and testing set according to the ratio of 6:2:2.

#### 2.2.2. ECG Detection

**Figure 1:**
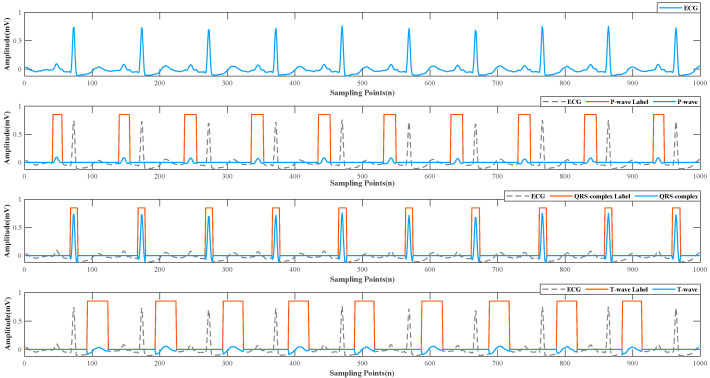
Raw ECG and P wave, QRS complex, T wave.

An ECG normal cardiac cycle contains three essential components: P wave, QRS complex, and T wave, representing different stages of cardiac activity. P wave is the first upward wave, which reflects the process of atrial depolarization and is an important detail in the diagnosis of arrhythmia. QRS complex is the most significant part, which is formed by ventricular contraction. T wave is the slow waveform that appears later, with a longer duration and gentle fluctuations representing the recovery process of ventricular excitation. Guillermo [36] applied the U-net architecture [37], which has been successfully applied in the field of biomedical image segmentation, for ECG delineation and achieved excellent results compared with traditional DSP methods. We adopted this architecture to extract three waveforms. Because the database in paper [36] is different from that of this paper, the accuracy of detection results cannot be guaranteed. However, the delineation results have a direct impact on the subsequent experiments, so we adopt a machine-based and manual-assisted strategy to manually verify the results of network segmentation. Because in an ECG cycle, the three characteristic waveforms have standard intervals, the automatic segmentation and manual inspection can reduce the workload and ensure the accuracy of the results. The extracted results of each ECG signal fragment are shown in Figure 1.

### 2.3. Multiple Regression Models

PPG has been widely used in BP prediction models. In order to verify the improvement of ECG waveform characteristics, we used five combinations as the input of the network: (1) PPG; (2) PPG + P wave; (3) PPG + QRS wave; (4) PPG + T wave; and (5) PPG + ECG. Meanwhile, to avoid accidental errors caused by a single model, we explored several well-known CNN architectures in our experiments, detailed as follows:

(1) AlexNet [38]: was one of the classic CNN networks in the past decade, which has laid an important foundation for the network and proved the effectiveness of CNN in complex models.
(2) GoogLeNet [39]: multiple convolutions or pooling operations were assembled into an inception module, and the network structure was constructed with this module. Then the network was concentrated in feature dimension, which reduced the number of parameters and further improved the network performance.
(3) ResNet18 [40]: proposed in 2015. It established the “shortcut connection” between the front and the back layer, which is useful for the back-propagation of gradients in the training process and deepens the number of layers.
(4) DenseNet121 [41]: established a dense connection between all previous layers and the back layer and realizes feature reuse by connecting features on the channel.
(5) DPN68 [42]: combined the fundamental ideas of ResNet and DenseNet. ResNet was the main framework to ensure low redundancy of features and a DenseNet branch was added to generate new features.

These original networks all take 2-dimensional images as input, while our experimental data are 1-dimensional signals. Therefore, we modified the first parameter of the convolution kernel to 1, so that the network structure could adapt to the experimental data. Other network parameters are not modified. The experiments were performed on a computer with 1 CPU at 2.6 GHz, 1 NVIDIA GeForce RTX2060 GPU and 64-Gb memory. All the models are run over highly efficient GPU using the PyTorch deep learning framework

### 2.4. Visualization with Grad-CAM

As a classification localization technology, Grad-CAM [27] can generate visual interpretation from any CNN-based network without an attention mechanism. Therefore, we can visualize the location of feature maps for these five models. For Grad-CAM, it uses the gradient to calculate the fusion weights of target feature images. The activation function Relu is then added to eliminate negative values and retain only those that have a positive effect on the results. As expressed by (1) and (2), A represents a feature map of size *i*, *j*, and dimension *k*. Then the importance weights are achieved by a global average pooling on *i* and *j*. Similar to CAM, it is possible to get $L_{\left(Grad-CAM\right)}^{c}$ by weighted sum.

(1) $$\alpha_{k}^{c}=\frac{1}{Z}\sum_{i}\sum_{j}\frac{\partial y^{c}}{\partial A_{ij}^{k}}$$

(2) LGrad−CAMc=ReLU(∑kαkcAk)

**Figure 2:**
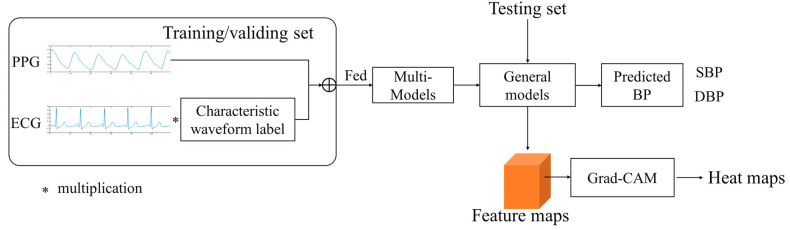
The overall framework of the experimental method.

For the selected five CNN models, we use the last convolution output before the fully connected layer as the input of Grad-CAM, and the output of Grad-CAM is a heat map of hot regions of a specific class that has the same size to the input signal [29]. The overall experimental process is shown in Figure 2.

## 3. Results

### 3.1. Metrics

In this study, the quantitative indicators for the performance evaluation of the blood pressure estimation model include the following [35,43]: mean error (*ME*), mean absolute error (*MAE*), standard deviation (*SD*), and root mean square error (*RMSE*). *ME* can reflect the relationship between the predicted value and the actual value. *SD* can reflect the dispersion degree of the prediction results. *MAE* and *RMSE* are also two important scales for evaluating models in machine learning. Compared with *MAE*, *RMSE* is more sensitive to outliers. These four indices are all expected to be as small as possible. Among them, *N* is the number of test segments, ${\hat{y}}_{}^{i}$, $y_{}^{i}$, and ${\bar{y}}_{}^{i}$ represent the predicted BP, reference BP, and average BP, respectively.

(3) $$ME=\frac{1}{N}\sum_{i}^{N}\left({\hat{y}}^{i}-y^{i}\right)$$

(4) $$MAE=\frac{1}{N}\sum_{i}^{N}\left|{\hat{y}}^{i}-y^{i}\right|$$

(5) $$SD=\sqrt{\frac{1}{N}\sum_{i}^{N}{\left({\hat{y}}_{}^{i}-\bar{y}\right)}^{2}}$$

(6) $$RMSE=\sqrt{\frac{1}{N}\sum_{i}^{N}{\left({\hat{y}}^{i}-y^{i}\right)}^{2}}$$

### 3.2. Analysis of Error Distribution

**Figure 3:**
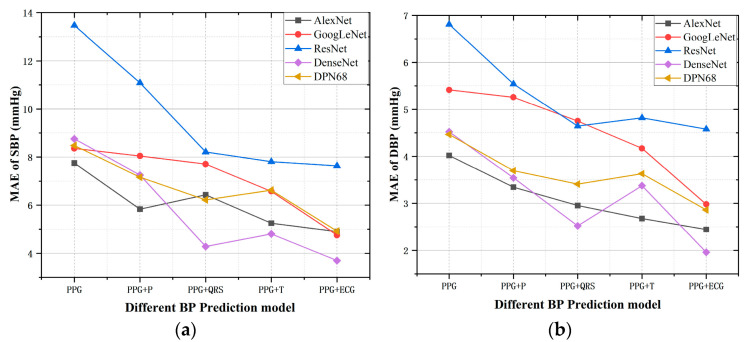
Estimation errors of five models with different input signal combinations. (**a**) MAE of SBP (**b**) MAE of DBP.

Table 1 presents the numerical comparison results of the mentioned five models with different signal combinations. Figure 3 compares the MAE estimated by different models for SBP and DBP under the five signal combinations. It can be found that the performance of these five CNN models was basically consistent, and the combination of PPG and ECG achieved the best results. This result is consistent with [20] that ECG contains important information related to BP, because the formation of BP is related to cardiac contractility, blood volume, and peripheral blood resistance. As an external manifestation of electrical signals of cardiac activity, ECG can be used to assess cardiac contractility, thus significantly improving the accuracy of blood pressure models.

For the specific three ECG characteristic waveforms, no matter which one was added, it would have a positive effect on BP prediction, which is more obvious in Figure 3. Generally, the P wave contributes the least to the accuracy of the model, and the QRS complex and T wave have relatively high contributions. In the QRS complex, the R-wave morphology can reflect the intensity of ventricular contraction and then affect cardiac ejection. The contribution of the T wave to the results was unexpected; it followed the QRS complex with a small amplitude but the longest duration of the three characteristic waveforms. This enlightens us that we can pay more attention to these two characteristic waveforms when performing feature extraction.

**Figure 4:**
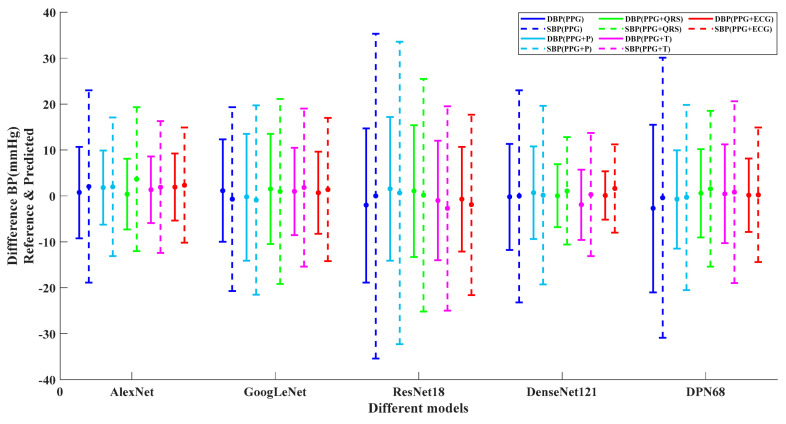
Mean and 95% CI plot for Different Models (solid dots represents mean value and short solid lines represent mean ± 1.96SD).

Figure 4 is a Bland–Altman plot established between the estimated and reference values in the testing group. The mean and limits of agreement (mean ± 1.96SD) are illustrated with the solid dots and short solid lines. The error distribution range with PPG and ECG as inputs is more concentrated. By adding ECG characteristic waveforms, the error distribution range becomes obviously more concentrated and reaches an optimum when PPG and ECG are used as inputs. This result is consistent in all five models.

### 3.3. Performance of Visualization

**Figure 5:**
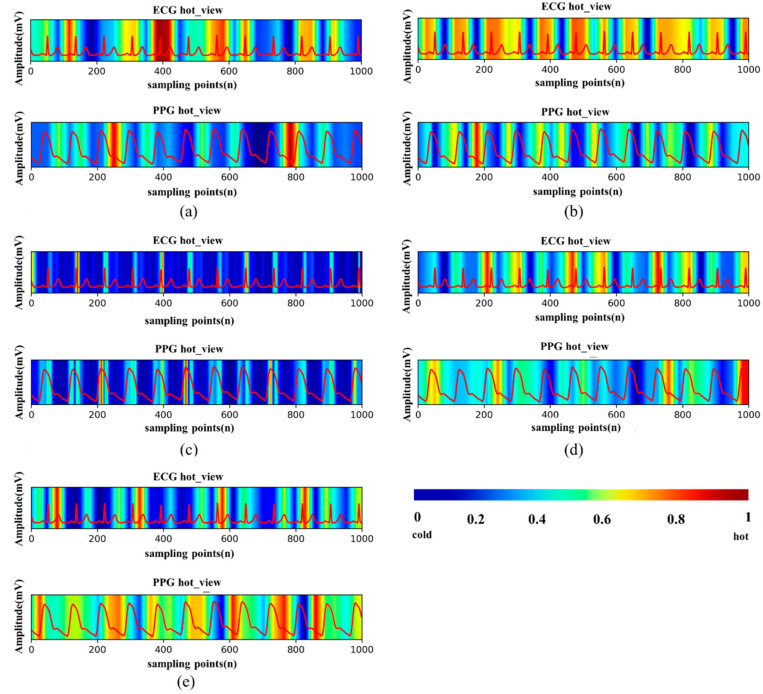
Heat maps generated by different models. (**a**) AlexNet. (**b**) GoogLeNet. (**c**) ResNet18. (**d**) DenseNet121. (**e**) DPN68.

Figure 5 shows the heat map of the features that each model learns when PPG and ECG are used as the signal input, in which the warm-colored parts indicate the more attention, and the cool-colored parts represent less attention on signal segments. For the ECG signal, it can be found that the networks show different degrees of highlight for each wave group. In Figure 5a,b,d,e, QRS and T waves receive more attention than P waves. Especially in Figure 5c, there is a significant periodic highlight in the QRS wave. This can be consistent with the results in Figure 3. Adding QRS and T waves greatly improves the accuracy of the prediction results because the network has learned more features in these two parts.

For PPG signal, we find that CNN networks are more likely to focus on the locations of peaks, valleys, and dicrotic notch, which are all the extreme points in a cycle of PPG. In traditional manual extraction of features [15,16,17,18], these points represent much time information and amplitude features, and scholars have deeply excavated them in both the time and frequency domains. This indicates that the features learned by the network inherit the advantages of artificial features.

It can be seen from Figure 3 that the prediction error of model Resnet18 is much larger than that of the other models when PPG and ECG are used as input signals. By comparing Figure 5c and others, we find that the focus of model Resnet18 is limited to the QRS complex, ignoring the rest waveform features; the characteristics learned in PPG are not significant, with only a narrow highlight at the peaks.

## 4. Discussion

Literature [44] has proved that ECG signals can be used to evaluate the contractility of the heart, which has a direct impact on the formation of blood pressure. The external manifestation of cardiac activity is the ECG signal, so the study of ECG waveform has an important role in predicting blood pressure. In the past, the blood pressure prediction models were mainly based on PPG, supplemented by ECG, or the whole ECG waveform as input, without specific analysis of the influence of the three characteristic waveforms of ECG on blood pressure prediction. In this paper, two experimental methods are used to get a consistent conclusion: QRS complex and T wave have a better effect on BP than P wave. In the first method, different ECG characteristic waveforms are combined to evaluate the results directly. In the second method, we try to explain the black box of CNN network with the use of Grad-CAM.

The experimental results may be due to the following two reasons. As can be seen from Figure 1, the QRS complex is the most prominent characteristic waveform during the ECG cycle [36,45]. Its amplitude fluctuates the most, and the amount of information it can provide is higher than that of the P and T waves with smaller amplitude changes. The addition of T wave also significantly improved the results, because the T wave lasts the longest and accounts for a larger proportion in a cycle. This experimental result shows that we can focus on these two waveforms if we need the most effective feature extraction for ECG in the blood pressure regression task. These results suggest that we should focus on these two waveforms when we need to extract features most efficiently or add attention mechanisms to the ECG in blood pressure regression tasks.

## 5. Conclusions

In this study, to explore the importance of the ECG characteristic waveforms in the BP estimation model, we used a common ECG detection procedure supplemented by manual annotation and divided the ECG signal into three parts: P wave, QRS complex, and T wave. Then we adopted five CNN models with multi-combination of signals as input. The results showed that compared with the single PPG, adding ECG waveforms can effectively improve the accuracy and the contribution of QRS and T wave is better than that of P wave. Meanwhile, we introduced the Grad-CAM to generate heat maps. Comparing the heat maps of different models, we found that the networks have a response to almost all segments of the ECG but the QRS and T waves received more highlights. This means that the network has learned more valuable features at these two waveforms. At the same time, if the network focuses on too one-sided parts, it is not conducive to the final result.

Extracting multiple features from an entire ECG may be a bit blind, but this article points out important directions: QRS and T waves. In future work, based on the information contained in the ECG signal, we will try to extract several effective artificial features; QRS and T wave will be the focus of research. Then they will be combined with PPG features to build a BP regression model. Attention mechanisms or other modules can also be added to the structure to take advantage of the CNN networks feature mining.

## Figures and Tables

**Table 1:** MAE and RMSE of five models with different input signal combinations.

**Models**	**AlexNet**				**GoogLeNet**				**ResNet18**			
	**SBP/mmHg**		**DBP/mmHg**		**SBP/mmHg**		**DBP/mmHg**		**SBP/mmHg**		**DBP/mmHg**	
	**MAE**	**RMSE**	**MAE**	**RMSE**	**MAE**	**RMSE**	**MAE**	**RMSE**	**MAE**	**RMSE**	**MAE**	**RMSE**
PPG	7.74	10.53	4.02	5.19	8.35	11.15	5.25	6.71	13.46	17.53	6.81	8.47
PPG + P wave	5.83	7.98	3.34	4.50	8.04	10.57	5.41	7.06	11.08	14.35	5.54	7.16
PPG + QRS wave	6.42	8.80	2.95	3.95	7.70	10.33	4.75	6.34	8.21	10.74	4.64	5.91
PPG + T wave	5.25	6.96	2.67	3.47	6.57	8.68	4.17	5.38	7.80	10.39	4.81	5.87
PPG + ECG	**4.93**	**6.2**	**2.44**	**4.19**	**4.75**	**6.44**	**2.97**	**3.97**	**7.63**	**10.26**	**4.58**	**6.21**
**Models**	**DenseNet121**				**DPN68**							
	**SBP/mmHg**		**DBP/mmHg**		**SBP/mmHg**		**DBP/mmHg**					
	**MAE**	**RMSE**	**MAE**	**RMSE**	**MAE**	**RMSE**	**MAE**	**RMSE**				
PPG	8.75	11.81	4.52	5.91	8.48	11.41	4.46	5.98				
PPG + P wave	7.25	9.80	3.54	4.71	7.16	9.64	3.69	4.95				
PPG + QRS wave	7.70	10.33	4.75	6.34	6.22	8.50	3.40	4.55				
PPG + T wave	4.80	6.87	3.37	4.35	6.62	9.14	3.62	4.90				
PPG + ECG	**3.69**	**5.18**	**1.95**	**2.71**	**4.92**	**6.86**	**2.86**	**3.97**				

## Data Availability

The data presented in this study are available from the corresponding author upon reasonable request.
